# Effects of mothers' socio-economic status on the management of febrile conditions in their under five children in a resource limited setting

**DOI:** 10.1186/1472-698X-6-1

**Published:** 2006-01-20

**Authors:** Adenike AE Olaogun, Abayomi A Adebayo, Olufemi E Ayandiran, Olayinka A Olasode

**Affiliations:** 1 Department of Nursing Science, College of Health Sciences, Obafemi Awolowo University, Ile Ife, Osun State, Nigeria; 2 Department of Economics, Faculty of Social Sciences, Obafemi Awolowo University, Ile Ife, Osun State Nigeria; 3 Department Of Dermatology & Venereology, College of Health Sciences, Ile Ife, Osun State, Nigeria

## Abstract

**Background:**

Public health research is shifting focus to the role of socioeconomic indicators in the promotion of health. As such an understanding of the roles that socio-economic factors play in improving health and health-seeking behaviour is important for public health policy. This is because the share of resources devoted to different policy options should depend on their relative effectiveness.

**Objective:**

To measure the effect of socio-economic status (age, education, occupation, income, religion and family structure) of mothers on the management of febrile conditions in under-fives children

**Method:**

Two hundred mothers who brought their febrile under-five children to a health facility were interviewed on the treatment they gave to their children before reporting at health facility. Data collected were entered and analyzed using the SPSS software. Binary logistic regression was adopted for the quantitative analysis of the effect of socio-economic variables on the mothers' actions prior to utilizing the health facility.

**Results:**

Results showed that while mothers' age was negatively correlated (-0.13), occupation was positively correlated (0.17) with under-fives mothers' action. Education, religion, income and family structure were however insignificant at 5% level

**Conclusion:**

This poses a lot of challenges to policy makers in the developing nations where women's education and earning capacity is low. There is therefore a need to increase the number of women benefiting from micro credit. This will ensure that more women are engaged in a form of occupation that is profitable and can sustain the economic and health needs of the family.

## Background

Access to health services and the quality of care administered at all levels of health care have been considered as the central determinants of health outcomes [1]. Recently, in public health research, the focus is shifting to the role of socio-economic indicators in the promotion of health [2]. There have been a lot of debates on the effects of socio economic factors on health outcomes. Mckeown [3] and Fogel [4] for instance submitted that improvements in longevity experienced in the 19th century in the Western world could be attributed more to improvement in nutrition consequent on higher income than to medical advances or public health campaigns. Others [5-9] have argued that these improvements were more on public health efforts (namely, sanitation, vaccination, and vector control) and advances in health technology such as discovery of more potent antibiotics than on income or income growth.

Szreter in a critique of McKeown's work posited that the return to generally declining mortality in the last third of the nineteenth century reflects the chronology of the most significant improvements in public health and urban sanitation rather than economic growth, rising living standards, and improved nutrition [7]. According to Szreter, that era witnessed the establishment of the Local Government Board; the passing of a series of Public Health Acts; and the implementation of a wide range of preventive measures that included supply of safe water, enforcement of environmental sanitation, and prevention of overcrowding [7]. However persuasive and academically sound Szreter's argument is, it leaves open the question of an indirect relationship between sanitary measures and improved nutritional efficiency [10]. It equally leaves open the question of a possible relationship between economic growth and the execution of preventive public health services.

Case [2] in a more recent work however observed that income exerts a causal effect on health status through several channels, among which she named improved nutritional status, better sanitation, improved living standards, reduction of psychological stress and reduced susceptibility to infections. She stated further that higher income might allow people to spend more time and money seeking out health services for self and household members. The study by Filmer, et al [11] further strengthens the case for quantifying the causal impact of income on health outcomes.

Other indicators of social economic status of a people are: educational attainment, nature of occupation, family background, and nature of property ownership and wealth. According to Hobbs and Blanks, occupation plays a major role in shaping the life style of individuals [12]. They asserted that there are correlations to be made between one's occupation and one's education, recreational and leisure-time activities, political affiliation and quality of basic social services. Indeed formal education has become a means to elevate one's social status and economic welfare. As such an understanding of the roles that socio-economic factors play in improving health and health-seeking behavior is important for public health policy. This has become imperative because the share of resources devoted to different policy options should depend on their relative effectiveness. Whatever the focus, there is a need to explore the role of socio economic factors on health outcomes particularly in a developing nation like Nigeria facing gross economic crises.

It is significant to note that most studies on the link of socio economic factors and health outcomes in the developing nations have focused on infant mortality [13]. In the United Nations' report for instance, mortality rate amongst the under fives in Nigeria was put at 178 per 1000 [14]. A major cause of this mortality is febrile conditions, which are not only preventable but also curable provided treatments are sought promptly and from appropriate centers. The term febrile condition refers to a state of being feverish which is usually associated with malaria, measles, neonatal tetanus, pneumonia, whooping cough etc [15,16]. Therefore, a case is being made for quantifying the causal impact that socio-economic indicators will have on health outcomes and health seeking behavior. Consequently, this paper analyses the effect of mothers' socio-economic status on the management of febrile conditions among their under-five children.

## Methods

A health facility based study was conducted in Modakeke, Ile -Ife in Osun State of Nigeria with the aid of interviewer-administered structured questionnaires. In line with the free health policy of the state government at the time of interview, health services were offered free of charge to the respondents captured in this interview. Coincidentally, this community had within the last two decades suffered from a prolonged communal crisis that resulted in loss of lives, property and relocation of residents to neighboring communities. As part of the government's effort in resettling the people in the intermittent periods of peace, a storey building was acquired and converted to a hospital. All these have combined to make the research setting a resource limited community.

A total of 200 mothers were selected by purposive sampling from all mothers who brought their children for treatment at the out patient department of the hospital. Inclusion criteria were: The respondents were biological mothers of sick children; the children had a febrile condition and were below 5 years of age. Data collection was done using interviewer-administered questionnaire. Apart from raising questions on the socio-demographic data of the respondents, the interviewer-administered questionnaire sought mothers' action or inaction prior to reporting at the hospital and the nature of such action. The questionnaire was however translated into Yoruba language (the local language of the people) before actual data collection.

Two female interviewers who were polytechnic students but natives of the community of study were trained in the use of the questionnaires. These interviewers were deliberately chosen from the community to facilitate the flow of information and all interviews were done on 'one on one' basis. The interviews were conducted in English and Yoruba language depending on the level of education of the respondents.

The information obtained from the questionnaires were coded and processed with SPSS version 11 software packages. Binary logistic regression was adopted for the quantitative analysis of the effect of socio-economic variables (age, income, education, occupation, religion and family structure) on the mothers' actions prior to utilizing the health facility. Logistic regression is particularly relevant because the dependent variable is binary (mother's action or inaction when children had febrile condition).

### Ethical Consideration

The community of study was visited and permission was sought from the community leaders. A letter was written to the Medical director of the only Government Health facility in the community seeking permission to use the facility as the base for the study. Verbal consents were obtained from each respondent prior to the interview and only those willing to participate in the study were included in the sample.

## Results

The socio-economic characteristics of the mothers are reflected in table 1. The ages of the mothers ranged from 15 years to 50 years with a mean of 26.2 and a standard deviation of 6.1. Majority of the mothers (66.6%) were in the young/middle adulthood age (21–30 yrs). The ages of their children ranged from 6 months to 5 years with a mean age of 2.1 years and the duration of child's illness ranged from 1 to 60 days with a mean of 4.8 days. The family structure revealed that majority (89.5%) came from monogamous homes. There is a general low literacy level as only 22% of the mothers had post secondary education. The occupational profile showed that over half – 115 (57.5%) were traders, 46 (23%) were artisans, 25 (12.5%) were full time housewives while the rest were teachers, nurses and other categories of civil servants (14%). Their income (neto and in kind) ranged from Nigerian Naira (N) 1 to 125, 000.00 (<1 US Cent – US$ 862.06) per week with a mean of N10, 000.00 (US$ 68.97) and a median of N6, 250.00 (US$ 43.1). About 70% of them earn below US$ 68.97 per week, 28% earn between US$ 68.97 to US$344.8 and the rest (2%) above $344.8 per week.

**Table 1:** Socio-Demographic Characteristics of Subjects

**Variables**	**Frequencies**	**Percentages**
**Age in years**		
15 – 20	31	15.5
21 – 25	67	33.3
26 – 30	67	33.3
31 – 35	22	11
36 – 40	6	3
45 – 50	7	3.5
**Total**	**200**	**100**
		
**Education**		
Primary Education	24	12
Secondary Education	133	66.5
Post Secondary Education	43	22
**Total**	**200**	**100**
		
**Family Structure**		
Monogamous	126	63
Polygynous	64	32
Single	10	5
**Total**	**200**	**100**
		
**Occupation**		
Traders	115	57.5
Artisans	46	23
Full time housewives	25	12.5
Civil Servants	14	7
**Total**	**200**	**100**
		
**Income in Naira(Neto & In-Kind)**		
Less than N10, 000.00	137	68.5
N10, 000.00 – N50, 000.00	56	28
Over N50, 000.00	7	3.5
**Total**	**200**	**100**

As depicted on table 2, a total of 162 (81%) mothers took an action before bringing child to clinic. On the nature of action taken, 129 (79.5%) gave orthodox drugs at home, 19 (11.25%) combined orthodox drugs with traditional herbs, while 7 (5.42%) used local herbs alone. Amount spent by mothers on drugs ranged from N0.00 to N 500.00 (US$ 3.45) with a mean of N82.49 (US$ 0.57). On the source of drugs, 50 (31.25%) bought them at the chemist, 98 (61.25%) bought them at the patent medicine stores while 10 (6.25%) obtained them at private clinics.

**Table 2:** Mothers' Response / Management of Sick Child

**Variable**	**Frequency**	**Percentage**
***Was an action taken?***	**(N = 200)**	
Took an Action	162	81
Did not take an action	38	19
**Total**	**200**	**100**
***Nature of Action Taken***	**(N= 162)**	
Used drugs at home	129	79.6
Used local herbs alone	7	5.42
Combined both traditional and orthodox	19	11.72
Sponge with water	5	1.8
Took child to private clinic	2	1.56
**Total**	**162**	**100**
***Source of Drugs***	**(N = 160)**	
Patent Medcine Store	98	61.25
Chemist	50	31.25
Private clinic	2	1.25
Home	10	6.25
**Total**	**160**	**100**

Using binary logistic regression, six socio-economic variables were considered as independent variables to capture the status of the under-five mothers. These independent variables were regressed on the action or inaction of the mothers (dependent variable). The result is as presented on table 3. As reflected on the table, the binary logistic regression result shows that age and occupation were accepted in the analysis as significant at less than 5% level while income, education, religion and family structure were not significant. This result implies that action or inaction of under-five mothers depend on age after controlling for occupation, income, education, religion, and family structure. And in reference to occupation, the result shows that action or inaction of under-five mothers depends on occupation after controlling for age, income, education, religion, and family structure. The **B **values as depicted on table 3 reveals that age is inversely correlated **(-0.13) **with the under-fives mothers' action when children have febrile illnesses while occupation exhibited a positive correlation **(0.17)**.

**Table 3:** Binary Logistic Regression Analysis of the Socio-Economic Determinants of Management of Febrile Conditions by Mothers of Under-Five (Variables in the equation)

**Variables**	**B**	**S.E.**	**Sig.**
**Age**	-0.13	0.05	0.01
**Occupation**	0.17	0.07	0.02
**Income**	0.00	0.00	0.31
**Education**	-0.09	0.19	0.65
**Religion**	-0.25	0.50	0.61
**Family Structure**	0.36	0.44	0.42
**Constant**	0.98	1.84	0.59

## Discussion

The community of study is located in the southwest Nigeria, a geo-political region that has been operating a free primary education policy from the 1950's for children that attend the public schools. Therefore it is not surprising that all the mothers had attended a form of formal education. However, due to the decline in the quality of education particularly in the public schools coupled with the high cost of tertiary education, most children could not proceed into tertiary institutions. This possibly explains why very few (22%) of the respondents had post-secondary education.

The study revealed that most of these mothers were engaged in self-employed occupations. This corroborates the Nigeria Demographic and Health Survey (NDHS) 1999 report in which over 70% of women working in the Southwest, are engaged in the informal setting. This pattern, to a large extent, is attributable to their low literacy level since education is a pre requisite for white collared jobs [17].

Contrary to the World Bank and the 2003 NDHS report that established a strong relationship between mothers' education and health seeking behaviour, this study finds no significant relationship between mothers' education and health seeking behaviour. The NDHS report particularly emphasized that the children of more educated mothers and those living in more economically advantage households are the least likely to experience febrile illnesses and that the likelihood of seeking treatment increase as education of mothers and economic index of household increases [18,19]. The observed incongruence cannot but be linked with the fact that the NDHS report is a national household survey while this study is a health facility based study conducted in one of the six geo-political regions in Nigeria. In a health facility based study as this, it is not likely that many of the highly educated mothers could be recruited; hence the insignificance of education in this study is traceable to the generally low educational level of most of the mothers interviewed.

As expected, occupation exhibited a positive relationship with health seeking action of the mothers. It is also recognized in literature that there is correlation between occupation and access to basic social services such as health, recreation and leisure activities [12,18] and [20]. Hobbs and Blanks [12] for instance submitted that occupation plays a major role in shaping life styles. According to them, it increases women's access to resources and strengthens their bargaining power within the household and the workplace. The women's access to resources and their bargaining power within the household have a significant influence on their treatment seeking behavior for their children [21].

The inverse relationship between age and under-fives mothers action when children develop febrile illness as observed in this study, indicates that the older the mothers are, the more likely they would not take an action when their under-fives develop febrile illness. This seeming lackadaisical response of the older women could be attributed to their previous experiences with children illness behaviour. The free medical care program of the ruling government in the area could explain the insignificance of income as reflected in this study as at the time of the survey. Hence the income of the mothers did not exhibit any influence on their management of their children's illness. Besides, the effect of income can be negative because, health services provision in such situation is not income determined. Consequently, the higher the income the higher the possibility of the rich neglecting the free medical service outlet for paid hospital. The rich may not be ready to endure the long queues that characterize public hospitals where free medical services are offered. Although Case (2002) reported that income exerts very strong effect on health status, however when health care services are provided to the public free of charge or subsidized, the effect of income may be weak or irrelevant to health seeking behaviour.

## Conclusion

The results of this study pose a lot of challenges to policy makers in the developing nations where the education of mothers are very low. There is a need to increase the number of women who are benefiting from micro credits. This will ensure that more women are engaged in a form of occupation that is profitable and can sustain the economic and health needs of the family.

## Competing interests

The author(s) declare that they have no competing interests.

## Authors' contributions

**AAEO **is the principal investigator. She conceived the study, was active at every stage of fieldwork, data analysis, and participated in the manuscript writing.

**AAA **mobilized the community, supervised the fieldwork, did the Econometric Analysis, and participated in the manuscript writing.

**OEA **reviewed the questionnaire, participated in the data analysis and contributed to the manuscript writing.

**OAO **participated in the questionnaire review, fieldwork supervision and result interpretation. All authors read and approve the submission of the manuscript.

## Pre-publication history

The pre-publication history for this paper can be accessed here:


